# Nexus Between Immune Responses and Oxidative Stress: The Role of Dietary Hydrolyzed Lignin in *ex vivo* Bovine Peripheral Blood Mononuclear Cell Response

**DOI:** 10.3389/fvets.2020.00009

**Published:** 2020-02-20

**Authors:** Maria Giovanna Ciliberti, Marzia Albenzio, Pasquale De Palo, Antonella Santillo, Mariangela Caroprese

**Affiliations:** ^1^Department of Sciences of Agriculture, Food and Environment, University of Foggia, Foggia, Italy; ^2^Department of Veterinary Medicine, University of Bari A. Moro, Bari, Italy

**Keywords:** ruminants, polyphenols, immune system, oxidative stress, cytokines, antioxidant response

## Abstract

The control of immune responses is particularly critical when an increase of oxidative stress occurs, causing an impairment of immune cell response and a condition of systemic inflammation, named oxinflammation. Nutritional strategies based on the use in the diet of phytochemicals extracted from plants, rich in antioxidants, could help restore the antioxidant/oxidant balance and obtain a modulation of immune response. Lignin represents a valuable resource of phenolic compounds, which are characterized by a corroborated antioxidant effect. To date, there are no studies reporting the effects of lignin in the diet on immune responses and oxidative stress in ruminants. The objective of the present experiment was the evaluation of the dietary inclusion of *Pinus taeda* hydrolyzed lignin on the *ex vivo* immune responses and oxidative stress biomarkers by peripheral blood mononuclear cells (PBMCs) isolated from beef steers. In order to test the effect during oxidative stress exposition, cells were treated with hydrogen peroxide (H_2_O_2_). The proliferation test and the viability assay were carried out on cells, whereas, on supernatants, the cytokine profile and the oxidative stress biomarkers were evaluated. The dietary inclusion with *P. taeda* hydrolyzed lignin resulted in cytoprotection after H_2_O_2_ exposition, increasing the number of viable monocytes and decreasing the reactive oxygen/nitrogen species production in supernatants. The cytokine profile indicated the modulatory role of hydrolyzed lignin on immune response, with a concomitant decrease of TNF-α and increase of IL-8 production, which are strictly connected with monocyte activation and antioxidant response pathway. These results demonstrated that hydrolyzed lignin may provide a modulation of oxidative stress and inflammatory response in PBMCs; thus, the *P. taeda* hydrolyzed lignin could be suggested as an innovative phytochemical in ruminant feed.

## Introduction

An inflammatory process can be originated by different stimuli that activate a physiological response with the aim of restoring homeostasis and controlling the internal constant milieu. The interconnected pathways of immune response, the innate and adaptive response, contributed to each other to protect the organism against pathogens or stressors capable of initiating an immune response. Moreover, when passing from innate to adaptive response, the interactions among phagocytic cells, T cells, dendritic cells (DCs), and regulatory T (Treg) cells are principally controlled by both intra- and extracellular redox environments (1). Under physiological conditions, the proper function of T cells is guaranteed by the equilibrium between reactive oxygen species (ROS) and antioxidant systems; thus, T cells displayed a controlled immune response. However, the alteration or accumulation of extracellular ROS and reactive nitrogen species (RNS) can alter the immune responses that can generate a systemic inflammatory status mainly induced by oxidative stress (OS) (2). Recently, the term “oxinflammation” has been introduced in order to explain the nexus that links chronic and systemic OS to mild chronic inflammation that negatively affects immune reactivity supporting adaptive responses and increasing the susceptibility to disease (3).

Nutritional strategies in animal feed including the supplementation of polyphenol-rich by-products could represent a model of circular economy to which the global market of food-feed production is oriented. Numerous phytochemicals have attracted scientific attention for their anti-inflammatory activity and modulating biological responses of the animals, as well as modulating rumen microbiome and improving meat/milk quality (4, 5). Interestingly, nutrigenomics study, both in human and in laboratory animals, demonstrated that nutraceutical compounds can affect gene expression, signaling processes, and other important processes, including cell apoptosis, immune modulation, and metabolism (6–9). A study on ovine neutrophils activated with phorbol myristate acetate (PMA) and treated with phytochemical extracts showed a modulation of their functions with an enhancement of anti-inflammatory effects by a reduction of cell adhesion and superoxide dismutase (SOD) production (10). Secretome can be defined by the secretory proteins from cells or organs, essential for signal transduction with specific membrane receptors and factors aiming at proliferation, growth, migration, and metabolism regulation (11). Cytokines are considered a key factor of the secretome; in particular, pro-inflammatory cytokines, such as tumor necrosis factor (TNF)-α, are able to activate the nuclear factor kappa-light-chain-enhancer pathway of activated B cells (NFκB) with enhancement of NADPH oxidase (NOX) activity in the mitochondria and increase of production of free radicals (12). Dietary phytochemicals from *Vaccinium myrtiullus* and *Curcuma longa* down-regulated mRNA expression of TNF-α and (NFκB) in sheep peripheral blood cells with a reduction of ceruloplasmin (13). Moreover, several aromatic plants and their secondary metabolites have been found to exert immunomodulatory actions when animals are immune-suppressed, improving phagocytosis, modulating immunoglobulin and cytokine secretion, enhancing lymphocyte expression, and boosting the release of interferon (IFN)-γ (14).

Supplementation with *Pinus taeda* hydrolized lignin (PTHL) of beef steers was found to be a proper additive for controlling methane production without any negative effects in terms of digestibility and production performance (5). Lignin represents a resource of phenolic compounds being transformed via hydroxyalkylation; furthermore, the application of phenolic resins from lignin is predicted to grow to 16 billion USD by 2025 (15) with a stable market value with a price of 1.22–1.46 USD/kg. Currently, phenols are produced from petroleum-based benzene by cumene process and are used in chemical, pharmaceutical, food, and perfumery industry (16). Phenolic compounds have largely demonstrated to have antioxidants and anti-inflammatory effects, in both *in vivo* and *in vitro* studies. No previous studies investigated the immunological effect of PTHL in ruminants. Our hypothesis was that dietary inclusion of lignin as phytochemical could improve the immune and antioxidant status of animals. Therefore, the present study aimed at understanding the role of PTHL on peripheral blood mononuclear cell (PBMC) proliferation, apoptosis, cytokine secretion, and antioxidant/oxidant balance.

## Materials and Methods

### Animals

The animal experiment was performed with approval from Ethics Committee for animal testing—CESA (process number 2-X/17). The experimental design was previously reported by Maggiolino et al. (5). Briefly, 40 Limousine steers (6 months old) were randomly subdivided into two groups; the experimental group received the supplementation with *P. taeda* hydrolized lignin (PTHL, initial mean live weight 339 kg, Oxyphenol®, I-Green, Padua, Italy), whereas the control (CON, initial mean live weight 340 kg) group did not receive supplementation. The experimental groups were fed *ad libitum* with the same total mixed ratio (TMR), for 120 days, until 10 months of age (final weight of 521 kg for PTHL group vs. 522 kg for CON). In particular, the PTHL group received 35 g/day per head for 90 days and 70 g/day per head for the last 30 days, administered directly in the mouth using a large syringe. The chemical composition of experimental diets, the PTHL composition, and its antioxidant activity were previously reported by Maggiolino et al. (5).

### Blood Samples and PBMC Isolation

At 120 days of the experiment, blood samples from animals were collected from the jugular vein into sterile vacuum tubes containing EDTA (Becton Dickinson). All experiments were performed using PBMCs obtained from 10 beef steers (*n* = 4 for the PTHL group, and *n* = 6 for the CON group).

PBMCs were isolated by Histopaque®-1077 density gradient (Sigma-Aldrich, Milan, Italy) according to Wattegedera et al. (17). Briefly, whole blood diluted 1:1 with cold PBS was centrifuged, and the white cell rings recovered after centrifugation were diluted in Hanks' balanced salt solution (HBSS) and slowly layered on the Histopaque®-1077 solution (10 ml). The tubes were centrifuged at 400 *g* for 30 min at 20°C, and the buffy coat, containing the PBMCs, layered on the upper layer of Ficoll-Paque, was recovered. The PBMC suspension was washed three times with HBSS wash buffer containing 2% fetal bovine serum (FBS), 50 μg/ml gentamicin (Sigma-Aldrich, Milan, Italy), and heparin (Sigma-Aldrich, Milan, Italy). Finally, PBMCs were resuspended in Iscove's Modified Dulbecco's medium (IMDM), without calcium and magnesium (Sigma-Aldrich, Milan, Italy) containing 10% FBS and 50 μg/ml gentamicin. PBMCs were counted in a Burker cell counting chamber, and the viability of cells was obtained by Trypan blue (>98%, Sigma-Aldrich, Milan, Italy) dye exclusion.

### Determination of Proliferation Assay and Viability

#### BrdU Assay

Cell suspension, containing 2 × 10^6^ cells/ml, was seeded into 96-well U-bottom plate into quadruplicate to carry out the proliferative response. PBMCs from the CON and PTHL group were stimulated with concanavalin A (ConA, at a final concentration of 5 μg/ml) and lipopolysaccharide (LPS, at a final concentration of 1 μg/ml). The concentration of both ConA and LPS used in *in vitro* trial was according to Ciliberti et al. (18). PBMCs stimulated with ConA and LPS were challenged with 4 mM (19) of H_2_O_2_ solution in the culture medium (OS H_2_O_2_-mediated) as OS inducer. PBMCs cultured in medium without stimulation represented the negative control. PBMCs stimulated with ConA and LPS represented the positive control. The plates were incubated at 37°C and 5% CO_2_ in a humidified incubator for 24 h. After incubation time, plates were centrifuged, and cell-free supernatants from each well were collected and stored at −20°C until ELISA to measure cytokine production and oxidant status.

After 24 h, a proliferation test was performed based on incorporation of bromodeoxyuridine (BrdU) in dividing cells using a commercial kit (Roche); briefly, BrdU is added to the cells and the cells were reincubated for 18 h; during this labeling period, the pyrimidine analog BrdU is incorporated in place of thymidine into the DNA of proliferating cells. Then, the culture medium was removed and the cells were fixed and subjected to DNA denaturation in one step. The BrdU incorporated during DNA synthesis was measured by ELISA using a monoclonal antibody from mouse–mouse hybrid cells (clone BMG 6H8, Fab fragments) conjugated with peroxidase and reading optical density with a plate reader spectrophotometer (Power Wave XS, Biotek, UK) at 450 nm was performed.

#### Evaluation of PBMC Viability by Fluorescent Imaging

PBMCs (1 × 10^6^ cells/ml) were seeded into 96-well flat-bottom plate into duplicate to determine the viability or cytotoxic effect of treatment on PBMCs (*n* = 2 CON group, *n* = 2 PTHL group). PBMCs were untreated (UNC), treated with LPS and ConA (SC, 1 μg/ml and 5 μg/ml, respectively), and with 4 mM of H_2_O_2_, and incubated for 24 h at 37°C/5% CO_2_. Then, PBMCs were stained with Image-iT® DEAD Green^TM^ viability stain (Molecular Probes, Milan, Italy) for 30 min, fixed, permeabilized according to the manufacturer's instruction, and imaged on Life Technologies EVOS™ XL Imaging System (Milan, Italy). The Image-iT® DEAD Green^TM^ is an impermeant dye when there is integrity of plasma membrane, whereas it is permeant when the integrity of membrane is compromised. The nucleus was detected with Hoechst 33342 in the DAPI/Hoechst channel, and the cell membrane permeability was detected in the FITC/GFP channel.

#### Assessment of Viability, Apoptosis, and Necrosis of PBMCs With Supravital Exposure to Propidium Iodide by Flow Cytometer

PBMC (1 × 10^6^ cells/ml) suspensions were added into polystyrene round bottom tubes for cytofluorimetric analysis and treated with LPS and ConA (1 and 5 μg/ml, respectively), and with 4 mM of H_2_O_2_ for 1 h. Then, supravital PI staining (Sigma-Aldrich, Milan, Italy, 50 μg/ml) on non-permeabilized cells for 30 min in the dark was performed according to Zamai et al. (20). The cells were washed with PBS and analyzed by flow cytometry. The positive control to PI was represented by cells treated with 0.2% Triton-X (diluted in PBS with 2% BSA). Negative control was represented by cells without PI staining. Samples were analyzed by Attune NxT Flow Cytometer (Thermo Fisher). FSC and SSC analysis were performed at each experiment for monocyte/lymphocyte gate strategy, and cell doublet exclusion was carried out by SSC-A and SSC-H gating. The number of events was stopped at 10,000 counts, and the absolute counts of monocyte/lymphocyte were used for the evaluation of their viability, apoptosis, and necrosis considering that apoptotic cells have decreased FSC.

### Determination of ROS Production, Total Antioxidant Capacity, and AOB Index in Supernatants

Relative levels of ROS in supernatants were measured according to the manufacturer's instructions of OxiSelect^TM^ *in vitro* ROS/RNS Assay Kit Green Fluorescence (Cell Biolabs Inc, ell Biolabs Inc., San Diego, CA) based on reaction of ROS and RNS species with 2′,7′-dichlorodihydrofluorescin (DCFH), which is rapidly oxidized to the highly fluorescent 2′,7′-dichlorodihydrofluorescein (DCF). Green fluorescence was read with a fluorescence plate reader at 480 nm excitation/530 nm emission (CLARIOstar microplate reader, BMG Labtech, Ortenberg, Germany). The fluorescence of blank samples was subtracted from sample measurements to eliminate background fluorescence. Results were read against DCF (1:10 scalar dilution with a concentration range of 0–10,000 nM) standard curve; the fluorescence intensity was directly proportional to the total ROS/RNS levels within the sample and was expressed as micromolar of DCF. Total antioxidant capacity (TAC) level in supernatants was evaluated using OxiSelect^TM^ TAC Assay Kit (Cell Biolabs, Inc), according to the manufacturer's instructions. Briefly, this assay is based on the reduction of copper (II) to copper (I) by antioxidants such as uric acid. The maximum absorbance at 490 nm was read against uric acid serial dilution standard curve (0–1 mM). The absorbance values were proportional to the sample's total reductive capacity and results were expressed as “μM of Uric Acid Equivalents,” proportional to the TAC of the samples. Both parameters of the redox balance were assessed together as the ratio of total antioxidant defenses and oxidants, namely, antioxidant oxidant balance (AOB), previously applied in Ciliberti et al. (21), where it was demonstrated a reliable index for the evaluation of the effects of diet on OS activated by heat stress exposition in sheep. Moreover, in human studies, it was applied in order to evaluate the antioxidant status of serum (22, 23) and after long-term intake of plant antioxidant-rich foods (24). An increase in the ratio indicates a lower risk for OS exposition due to a prompter antioxidant defense against pro-oxidant production.

### Determination of IL-10, IL-12, IL-8, IFN-γ, and TNF-α in Culture Supernatants by ELISA

The ELISA for IL-10 and IL-12 in PBMC supernatants was determined according to Kwong et al. (25) and Hope et al. (26), with some modifications as previously reported in Ciliberti et al. (27). The sandwich ELISA was built using specific antibody against bovine IL-10 and IL-12 (AbD Serotec, Kidlington, UK). The plates were read at 450 nm by a spectrophotometer (Power Wave XS, Biotek). Data were expressed as nanograms per milliliter for both IL-10 and IL-12. The intra-assay coefficients of variation (CV) were 9.46% for IL-10 and 10% for IL-12.

The IL-8, IFN-γ, and TNF-α in supernatants were assayed using 96-well plates (Sterilin, Cambridge, UK), coated overnight at 4°C with 100 μl of mouse anti-ovine interleukin-8 antibody (with checked bovine cross reaction, Kingfisher Biotech, St. Paul, MN), anti-bovine IFN-γ (AbD Serotec, Kidlington, UK), and anti-bovine TNF-α (AbD Serotec, Kidlington, UK) antibodies dissolved in PBS for IL-8 (2 μg/ml final concentration) and carbonate buffer pH 9.6 for IFN-γ (2 μg/ml, final concentration) and TNF-α (5 μg/ml, final concentration), respectively. After washing four times with PBST (PBS/Tween 20, 0.05%), the blocking solution including bovine serum albumin (PBST/BSA 3%) was added into wells for 1 h. Interleukin-8, IFN-γ, and TNF-α secretion in supernatants were calculated against a standard curve using recombinant protein specific for bovine, and the standards and supernatants were added into wells and incubated for 1 h; subsequently, the plates were washed four times. Biotinylated secondary anti-bovine IFN-γ antibody (2 μg/ml, final concentration, AbD Serotec) and anti-bovine TNF-α antibody (2.5 μg/ml, final concentration in PBS, Kingfisher Biotech) dissolved in PBS were added for 45 min. For the IL-8 antibody conjunction, the rabbit anti-sheep interleukin-8 (2 μg/ml, final concentration in PBS, AbD Serotec) was used and added to the wells for 1 h. After washing four times, 100 μl of streptavidin–horseradish peroxidase (HRP) (1/500, AbD Serotec) was added for IFN-γ and TNF-α assay, whereas, for IL-8, a goat anti-rabbit IgG HRP-conjugated antibody (1/20,000 in PBS, Sigma-Aldrich, Italy) was added into wells. After washing, 100 μl of 3,3′,5,5′-tetramethylbenzidine (TMB, Sigma-Aldrich, Milan, Italy) substrate solution was added to each well and the colorimetric reaction was stopped adding H_2_SO_4_ (1 M). All plates were read at 450 nm by a spectrophotometer (Power Wave XS, Biotek). Data were expressed as nanograms per milliliter for both IL-8 and TNF-α, and the level of IFN-γ was measured as picograms per milliliter. The intra-assay CV was around 10% for IL-8, IFN-γ, and TNF-α.

### Statistical Analysis

All data are presented as mean ± SEM. Data were checked for normality test and analyzed with mixed ANOVA model of SAS (28). The model included the fixed effects of *in vitro* treatment (UNC, SC, and H_2_O_2_), the feeding strategy (CON and PTHL), and their interaction. Animals are included in the model as random effect. The significance of the differences was assessed using Tukey *post hoc* test for multiple comparisons and the *P* value of <0.05 was considered statistically significant. Pearson correlation analysis was performed to correlate panel of cytokine, OS measurenments and proliferation of PBMCs.

## Results

### PBMC Proliferation and Viability by Fluorescent Imaging

**Figure 1:**
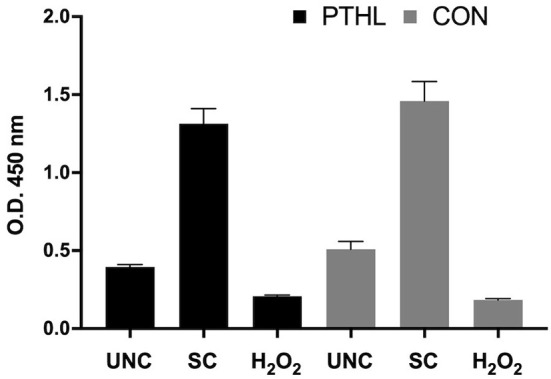
BrdU incorporation in proliferating PBMCs isolated by blood of beef steers supplemented with *Pinus taeda* hydrolyzed lignin (PTHL) and with a control diet (CON). Cells were unstimulated (USC), stimulated with LPS and ConA (SC, 1 and 5 μg/ml, respectively) and with 4 mM of H_2_O_2_, and incubated for 24 h at 37°C/5% CO_2_.

**Figure 2:**
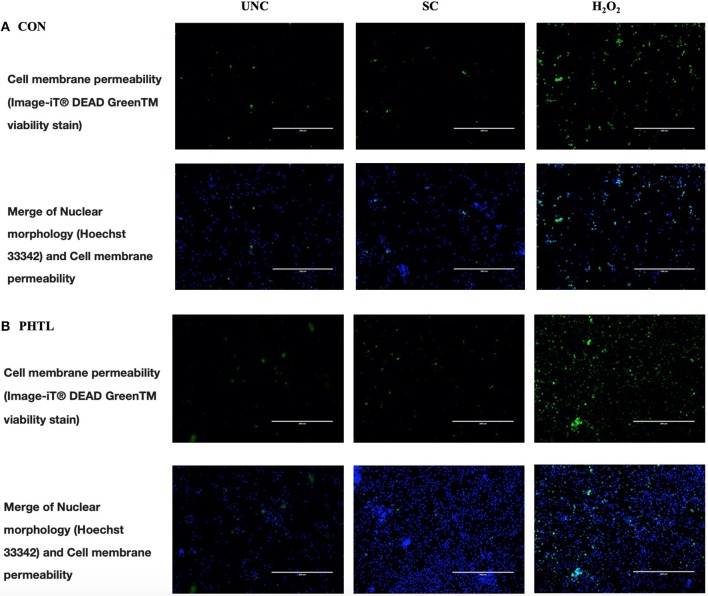
Imaging of PBMCs from the CON group **(A)** and from the PTHL group **(B)**, and viability was obtained using the Image-iT® DEAD GreenTM Viability Stain. PBMCs were untreated (UNC), treated with LPS and ConA (SC, 1 and 5 μg/ml, respectively) and with 4 mM of H_2_O_2_, and incubated for 24 h at 37°C/5% CO_2_. Then, PBMCs were stained with Image-iT® DEAD GreenTM viability stain for 30 min, fixed, permeabilized according to the manufacturer's instruction, and imaged on Life Technologies EVOS™ XL Imaging System (Milan, Italy). The nucleus was detected with Hoechst 33342 (blue) in the DAPI/Hoechst channel, and the cell membrane permeability was detected in the FITC/GFP channel (green).

The BrdU incorporation in dividing cells was influenced by the *in vitro* treatments (*P* < 0.0001) and by the interaction between *in vitro* treatment and feeding strategy (*P* = 0.0002). No significant differences were registered in PBMCs from the PTHL group and the CON group (Figure 1). The intense proliferative response of PBMCs and their viability emerged using Green Image-iT® DEAD Green^TM^ viability stain. In Figure 2, the membrane permeability (green) and its merge with nuclear morphology (blue) of PBMCs from the CON group (a) and the PTHL group (b) are represented. In UNC and in the H_2_O_2_ treatment, the viability clearly decreased with respect to SC in both CON and PTHL groups. However, in the PTHL group, the cell viability, as depicted from merging images from nuclear and membrane permeability (blue and green stain), was higher than in the CON group; this result was particularly apparent when comparing the viability of total cell number after OS H_2_O_2_-mediated treatment.

### Viability, Apoptosis, and Necrosis of PBMCs With Supravital Exposure to Propidium Iodide by Flow Cytometer

**Figure 3:**
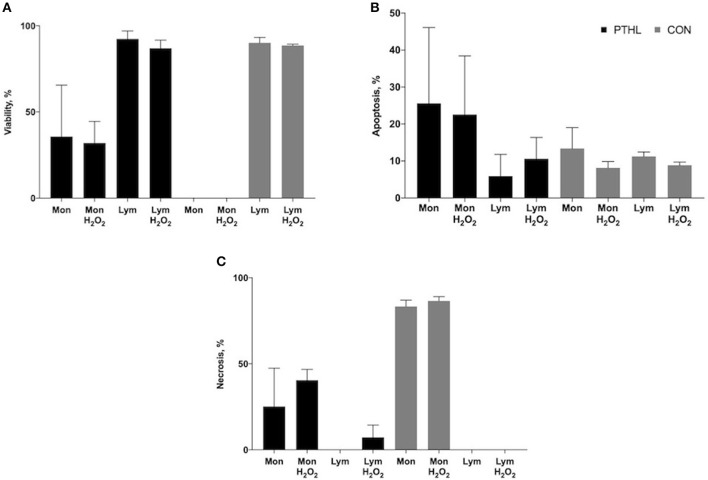
Percentage of viability **(A)**, apoptosis **(B)** and necrosis **(C)** of monocyte/lymphocyte stained with supravital exposure to propidium iodide (PI, 50 μg/mL). Cells (1 × 106) were pretreated with LPS and ConA (1 μg/mL and 5 μg/mL, respectively) and with H_2_O_2_ (4 mM) for 1h, then stained with PI for 30 min at the dark and washed before flow cytometric analysis. The supravital PI exposure on unfixed cells allowed to the simultaneous detection of living (PI negative) apoptotic (PI dim) and necrotic (PI bright) cells.

When measuring cell viability by flow cytometer, both monocyte and lymphocyte population characteristics of PBMCs can be discriminated by FSC and SSC analysis. Exposure to propidium iodide at supravital dose is a reliable method to concomitantly identify the detection of living (PI negative), apoptotic (PI dim), and necrotic (PI bright) cells. After gating of monocytes and lymphocytes, the percentages of viable (Figure 3A), apoptotic (Figure 3B), and necrotic (Figure 3C) cells of each population were measured in both the CON and PTHL group. After 1 h of H_2_O_2_ treatment, the lymphocytes in both the PTHL and CON group did not register a decrease in viability, whereas monocyte viability decreased in the CON group in comparison with the PTHL group (*P* = 0.03). As a consequence, in the CON group, the majority of monocyte population seemed to undergo necrosis, while in the PTHL group, the monocyte viability was around 35.7% without H_2_O_2_ treatment and 31.9% after 1 h of H_2_O_2_ treatment. Thus, results demonstrated that the lignin administration in the PTHL group was able to protect monocytes from necrosis.

### ROS/RNS Production, TAC, and AOB Index in Supernatants

**Figure 4:**
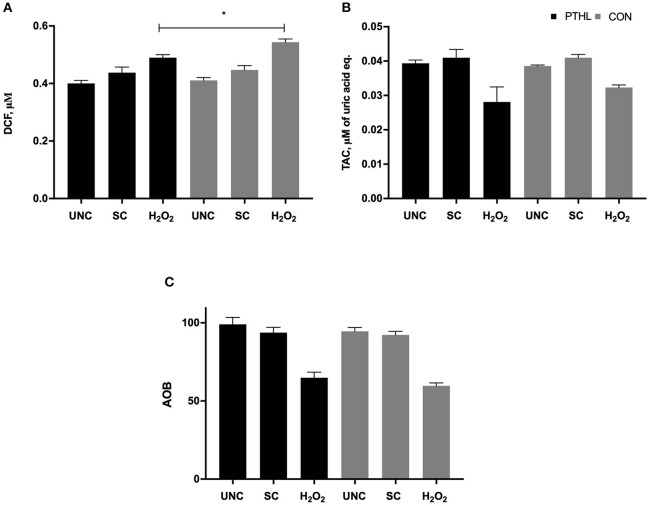
ROS/RNS production measured as micromolar of DCF **(A)**, total antioxidant capacity measured as micromolar of uric acid equivalents, **(B)** and Antioxidant/Oxidant Balance (AOB = TAC/ROS-RNS) **(C)** in supernatant of PBMCs unstimulated (UNC) and stimulated with LPS and ConA (SC, 1 and 5 μg/ml, respectively) and with 4 mM of H_2_O_2_ for 24 h at 37°C/5% CO_2_. *P < 0.05 were considered significant among feeding strategy.

Total ROS/RNS production in supernatants from PBMCs was influenced by feeding strategy (*P* = 0.03) and by *in vitro* stimulation (*P* < 0.0001). On average, ROS/RNS production was higher in the CON group than in the PTHL group. The PTHL group resulted in a lower ROS/RNS production than the CON group after H_2_O_2_ treatment, and as expected, the unstimulated cells of both groups showed lower ROS/RNS production than stimulated PBMCs (Figure 4A). The TAC significantly decreased when adding H_2_O_2_ to both PTHL and CON PBMCs (*P* < 0.0001). No significant differences in the TAC levels in supernatant between experimental groups were found (Figure 4B). When the AOB was calculated in order to verify the antioxidant status of PBMC supernatants, no differences between experimental groups, also during H_2_O_2_ exposition, were found (Figure 4C).

### Production of IL-10, IL-12, IL-8, IFN-γ, and TNF-α in PBMC Supernatants by ELISA

**Figure 5:**
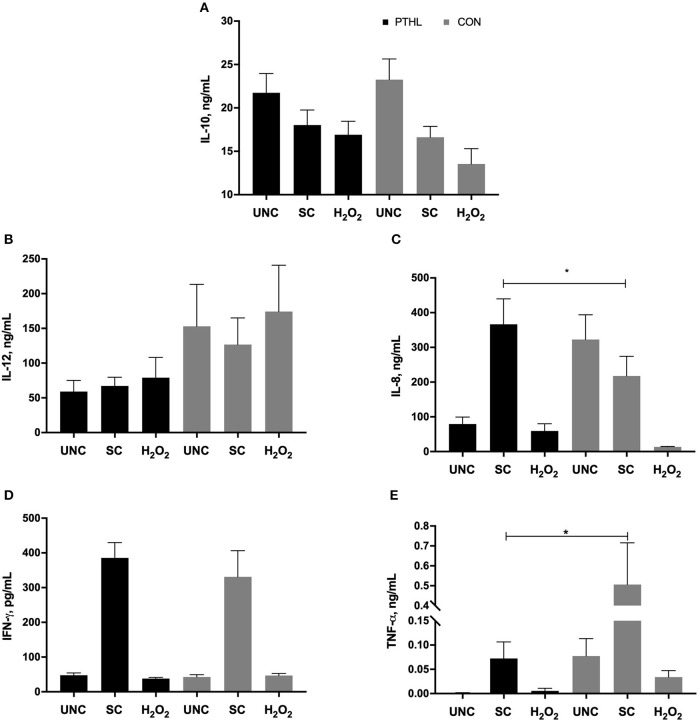
Interleukin (IL)-10 **(A)**, IL-12 **(B)**, IL-8 **(C)**, interferon-(IFN)-γ, **(D)**, tumor necrosis factor (TNF)-α, and **(E)** secretion by PBMCs unstimulated (UNC) and stimulated with LPS and ConA (SC, 1 and 5 μg/ml, respectively) and with 4 mM of H_2_O_2_ for 24 h at 37°C/5% CO_2_. *P < 0.05 were considered significant among feeding strategy.

As regards the cytokine secretion, the production of IL-10 by PBMCs was not affected by diet supplementation, registering a significant effect in relation to *in vitro* treatment (*P* = 0.0014). In particular, in the CON group, the H_2_O_2_ treatment resulted in a decrease of IL-10 production in comparison with IL-10 production from UNC PBMCs (*P* = 0.0076, Figure 5A), whereas the IL-12 production did not result in any significant differences among experimental diet and *in vitro* stimulation (Figure 5B). Furthermore, the *in vitro* treatment affected the IL-8 production (*P* < 0.0001), showing a significant interaction between diet supplementation and *in vitro* treatment (*P* = 0.0002). On average, the IL-8 production of SC was the highest followed by UNC and H_2_O_2_-treated PBMCs, respectively. Moreover, the SC PBMCs from the PTHL group produced higher IL-8 than SC PBMCs from the CON group (*P* = 0.049, Figure 5C).

The production of IFN-γ was significantly driven by *in vitro* stimulation (*P* < 0.0001); both UNC- and H_2_O_2_-treated PMBCs from the PTHL and CON group had lower IFN-γ production than SC PBMCs (Figure 5D). Finally, the TNF-α production in SC PBMCs from the PTHL group was significantly decreased with respect to SC PBMC from the CON group (*P* = 0.019, Figure 5E).

**Figure 6:**
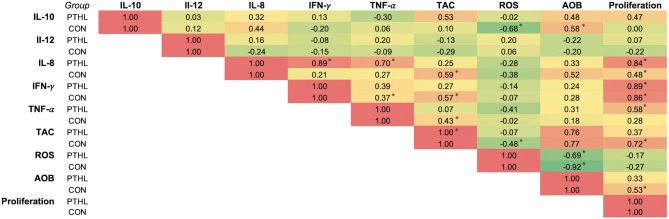
Correlation analysis of panel of cytokine, oxidative stress measurements, and cell proliferation of the CON and PTHL group. *P < 0.05.

Correlation coefficients (*r*) of the panel of cytokines, OS measurements, and proliferation of PBMCs from the PTHL and CON group were represented as heat map in Figure 6. In the PTHL group, IL-8 positively correlated with TNF-α, IFN-γ, and proliferation (*P* < 0.05); additionally, both TNF-α and IFN-γ correlated with proliferation (*P* < 0.05). In the CON group, IL-10 was found to be positively correlated with ROS and AOB; IL-8 correlated with TAC, ROS, AOB, and proliferation (*P* < 0.05).

## Discussion

The present study has provided evidence that *in vivo* supplementation with *P. taeda* hydrolyzed lignin (PTHL) of beef steers is able to influence the immune response, modifying cellular apoptosis/necrosis and viability, the oxidative status, and the cytokine profile of PBMCs treated with H_2_O_2_.

Hydrogen peroxide, being a major ROS, can be considered an activator of OS in different cellular models responsible for cell death and apoptotic variations (29–31). In the present paper, the inclusion of H_2_O_2_ treatment at 4 mM was used in order to cause OS, as previously reported in Asiaei et al. (19). Furthermore, *in vitro* investigations utilize mitogens, such as ConA and phytohemagglutinin, which can activate mammalian T-cell proliferation via mitosis stimulation (32, 33); thus, mitogens are used in defining the non-specific cellular immune response. Besides, blastogenic responses of bovine peripheral blood leukocytes stimulated with *E. coli* LPS, as an activator of bovine B-cell mitosis, are enhanced by immunization with heterologous bacteria (34). In a previous study, LPS stimulation was used for simulating an *in vitro* persistent inflammatory response causing a decreasing of cell viability and enhancement of inflammatory cytokine synthesis (35). The blastogenic response of bovine PBMCs to LPS *in vitro* was found to be correlated with disease resistance of periparturient dairy cows (36). In the present study, PBMCs isolated from blood of beef steers were stimulated with ConA and LPS in order to stimulate both T and B cells during OS exposition and evaluate a more comprehensive effect of PTHL on immune competence mediated by cytokine production.

The PTHL supplementation did not cause an enhancement of proliferative response to BrdU incorporation; however, the increased levels of proliferation in response to mitogens in both experimental groups demonstrated that *in vitro* proliferative induction could be representative of cellular immunocompetence (37). The blastogenic response to LPS has been previously considered as a useful prognostic parameter of the host's coping ability with respect to environmental, infectious, and non-infectious stressors (38). The OS *in vitro* treatment was not counterbalanced by PTHL *in vivo* supplementation as regards the proliferative response; however, hydrolyzed lignin contributed to determine a major viability of PBMCs as demonstrated by fluorescent imaging and flow cytometry assays. The hypothesis of a possible cytoprotective role of PTHL on PBMCs was suggested by the decrease of cell membrane permeability in PBMCs from the PTHL group detected by fluorescent microscopy. Indeed, when the viability was evaluated by flow cytometry, using the supravital dose of PI, the cytoprotective role of PTHL emerged, particularly on monocytes. The principle of cytometric analysis for detecting viability, apoptosis, and necrosis of cells is based on light scattering changes during apoptosis, with cells being smaller, and the intensity of side scatter diminished. In contrast, during necrosis, both light scatter parameters decreased, caused by loss of membrane permeability and leakage of cytoplasmic constituents (39). The protective role of hydrolyzed lignin against apoptosis and necrosis was emphasized by the presence of a percentage of viable monocytes also during OS H_2_O_2_-mediated *in vitro* treatment in the PTHL group. In contrast, the CON group did not register any viable monocytes, which are mainly gone due to the irreversible necrosis process. The plethora of different assays is strongly requested to cross-analyze the actions of PTHL on changing cell morphology given better information about the role of hydrolyzed lignin on PBMCs (40).

The composition of PTHL extract contained flavonoids and polyphenols that account for its total antioxidant activity. The PTHL supplementation contributed to reduce ROS/RNS production by PBMCs exposed to OS H_2_O_2_-mediated treatment; however, a slight reduction of TAC in both groups exposed to OS H_2_O_2_-mediated treatment as a response of counterbalancing of oxidant products was registered. The absence of an increase in TAC in the PTHL group could be explained by the dual role of flavonoids as antioxidant and pro-oxidant in relation to their concentration. Schmalhausen et al. (41) reported that the flavonoid quercetin at concentrations higher than 200 μM contributed to the formation of hydroxyl radical and production of H_2_O_2_, especially in the presence of metal ion; conversely, it protected from oxidation in the absence of a metal ion. Tea polyphenols tested in non-ruminant cells, both *in vitro* and *in vivo*, showed an antioxidant effect against ROS-induced disorders (42), directly by ROS scavenging and by stimulation of endogenous cellular defense systems (43). When H_2_O_2_ was tested at supraphysiologic dose in bovine mammary epithelial cells, the *in vitro* treatment with tea polyphenols stimulated the activity of antioxidant endogen enzymes, such as SOD and glutathione peroxidase (GSH-Px), and decreased ROS, malondialdehyde (MDA), protein carbonyl, 8-hydroxy-2′-deoxyguanosine, 8-isoprostaglandin, and the activity of caspase-3, reducing the damage of proteins, DNA, and lipids (44). It has been demonstrated that cell susceptibility to H_2_O_2_ is dose dependent; in the memory T cells, susceptibility to H_2_O_2_ is lower than in effector T cells and Treg cells, exploiting their suppressive action also in the presence of micromolar levels of H_2_O_2_ (1). Owing to the above, we can hypothesize that dietary antioxidants as well as PTHL extract can contribute to immune cellular activity mediating the apoptosis and ROS production during OS H_2_O_2_-mediated treatment.

Dietary antioxidants could modulate the inflammatory response with different mechanisms, via indirect control by antioxidant defense or interfering with the OS signaling; the direct action of dietary antioxidants on inflammation was the suppressive action on pro-inflammatory signaling transduction (45). Principally, it has been demonstrated that anti-oxidative activity and anti-inflammatory effects of phenolic compounds involve similar biomarkers. The key node that cross-links the signaling cascades between redox response and inflammation, caused by ROS overproduction at the mitochondrial level, was found to be the NLRP3 (NACHT, LRR, and PYD domain-containing protein 3) inflammasome assembly, which stimulates the synthesis of pro-inflammatory cytokines (46). The activated inflammasome, in a cascade mechanism, stimulates the release of cytokine IL-1β from cytoplasm into extracellular environment and activates Toll-like receptor (TRL)-1-mediated inflammatory signaling to produce IL-1β, IL-6, IL-8, TNF-α, and IFN-γ. The activation of these cascades of signals serves to amplify the inflammatory response that is responsible for systemic inflammation (45). In the present experiment, dietary PTHL altered the release of TNF-α in supernatants of PBMCs exposed to stimulation, revealing a strong control of inflammatory response due to antioxidant compounds. The pluripotent cytokine TNF-α is considered to be the endogenous mediator of inflammation activating the cytokine cascade required for appropriate cellular responses in the target tissue (47). Circulating monocytes are the main cellular target of TNF-α, which is responsible for the phenotypic changes required for phagocytosis process and the induction of secretion of other inflammatory mediators, such as IL-8, which allows for the recruitment of neutrophils into the target tissue (47). Moreover, IL-8 is an important inflammation mediator that can be induced by ROS and suppressed by antioxidants in a cell-type-specific fashion (48–50). Zhang et al. (51) demonstrated a novel paradigm in which the response of Nrf2 (NF-E2-related factor 2) to OS by inducing expression of cytoprotective and antioxidant genes that attenuate tissue injury, which is mediated by chemokine production and by an increase in IL-8 after an initial inflammatory response, reduces inflammation, and preserves tissue integrity. This result may be relevant for limiting tissue injury diseases in which OS and inflammation play a prominent pathophysiologic role and should be considered for therapeutic strategy. The use of PTHL in the diet of beef steers allowed the increase of IL-8 secretion in stimulated PBMCs compared to CON. Based on this, and according to an increase of viability of monocytes in PTHL PBMCs, the dietary inclusion of PTHL clearly showed a cytoprotective role and a control of inflammation mediated by a decrease of TNF-α secretion, the concomitant increase of IL-8 secretion, and the reduction of ROS production. The presence of apoptotic cells during monocyte activation influences cytokine patterns by increasing IL-10 production and reducing pro-inflammatory cytokines; however, the changes in cytokine patterns from cells depend on incubation time (52). In the present experiment, incubation times of cells in which apoptosis and necrosis were measured differed from those of cells in which cytokines were measured. Based on this, cytokine results cannot be strictly interrelated with results from necrosis and apoptosis assay.

There exist a bimodal regulation between inflammatory response and OS; thus, a lower OS reduces the production of inflammatory cytokines and, in turn, a decrease in inflammatory cytokines is capable of decreasing the production of free radicals (53). Moreover, the interrelation between the control of immune responses and PTHL dietary administration was corroborated by the significant correlations found among TNF-α, IL-8, IFN-γ, and PBMC proliferation, whereas the significant correlations found in the CON group deeply demonstrated an inflammatory profile of PBMCs, mediated by a cross-talk between OS and cytokine production in which IL-10 correlated with ROS and AOB and IL-8 correlated with TAC, ROS, AOB, and PBMC proliferation.

## Conclusions

In the present experiment, the effect of PTHL dietary supplementation on beef steers' immune cell function and cytokine profile during OS H_2_O_2_-mediated treatment was addressed. The use of PTHL, rich in hydrolyzed lignin with an antioxidant effect, was able to protect cells from apoptosis/necrosis, due to OS exposition, increasing the number of viable monocytes, and decreasing the ROS/RNS production in supernatants. Furthermore, a modulation of inflammatory responses of PTHL supplementation emerged in PMBCs with a decrease of TNF-α and an increase of IL-8 production, which are strictly connected with monocyte activation and antioxidant response pathway. Data of the present study improved knowledge about the use of hydrolyzed lignin in the diet of ruminant emphasizing its antioxidant and immunological role. Results encourage further experimentations about the potential application of hydrolyzed lignin in dairy animals under OS due to physiological events.

## Data Availability Statement

The datasets generated for this study are available on request to the corresponding author.

## Ethics Statement

The animal study was reviewed and approved by Ethics Committee for animal testing–CESA (Process number 2-X/17).

## Author Contributions

MC, MGC, and PD were involved in the original design of the study. PD was responsible for the on-farm trial. MGC, MA, and AS were responsible for analytical procedures and data analysis, and wrote the manuscript. All authors have read and approved the final version of the manuscript and contributed to the preparation of the manuscript.

### Conflict of Interest

The authors declare that the research was conducted in the absence of any commercial or financial relationships that could be construed as a potential conflict of interest.
